# Longitudinal [^18^F]LW223 PET imaging of macrophage-driven inflammation following myocardial infarction in a rat model: implications for left ventricular remodelling

**DOI:** 10.1007/s00259-025-07691-4

**Published:** 2025-12-12

**Authors:** Mark G. MacAskill, Victoria J. M. Reid, Carlos J. Alcaide-Corral, Timaeus E. F. Morgan, Lachlan Waddell, Adrian J. W. Thomson, Takeshi Fujisawa, Nicholas L. Mills, Judit A. Marti, Dominic Kurian, Thomas M. Wishart, Ana Clara Juan De Albuquerque, Ernest Chui, Agne Knyzeliene, Viktoria Balogh, Catriona Wimberley, Marc R. Dweck, David E. Newby, Christophe Lucatelli, Sally L. Pimlott, Andrew Sutherland, Adriana A. S. Tavares

**Affiliations:** 1 Center for Cardiovascular Science, QMRI, University of Edinburgh, 47 Little France Crescent, Edinburgh, EH16 4TJ UK; 2 Edinburgh Imaging, University of Edinburgh, Edinburgh, UK; 3 School of Chemistry, University of Glasgow, Glasgow, UK; 4 The Roslin Institute, University of Edinburgh, Edinburgh, UK; 5 Centre for Systems Health and Integrated Metabolic Research, Nottingham Trent University, Nottingham, UK; 6 Centre for Clinical Brain Sciences, University of Edinburgh, Edinburgh, UK; 7 Institute of Particle and Nuclear Physics, University of Edinburgh, Edinburgh, UK; 8 School of Medicine, University of Glasgow, Glasgow, UK; 9 NHS Greater Glasgow and Clyde, Glasgow, UK

**Keywords:** [^18^F]LW223, PET, Myocardial infarction, Inflammation, TSPO

## Abstract

**Purpose:**

Inflammation affects cardiac remodelling following myocardial infarction (MI), and can be imaged using Positron Emission Tomography (PET) targeting the 18 kDa translocator protein (TSPO). We utilised a rat reperfusion MI model to assess whether longitudinal [^18^F]LW223 could accurately measure macrophage-driven inflammation using outcome measures amenable to clinical translation, in addition to assessing the prognostic potential of [^18^F]LW223 for cardiac dysfunction.

**Methods:**

Adult male Sprague–Dawley rats underwent coronary artery ligation and reperfusion to induce MI. [^18^F]LW223 PET/Computed Tomography was performed longitudinally on day 2, 7, 14 and 28 post-MI. On day 28, cardiac function was assessed by ultrasound. Naïve and sham rat controls were compared to the MI cohort. A separate cohort of rats were produced for histological validation and proteomic analysis.

**Results:**

[^18^F]LW223 standard uptake value corrected for myocardial blood flow (*SUV*_*MBF*_) was highest within the MI cohort and localised to the infarct. This peaked at day 2 and remained elevated versus naïve and sham controls out to day 28. These patterns were validated by histology, revealing that the majority of TSPO expressing cells within the infarct at day 2 were also CD68^+^ (55.2%). Proteomics confirmed upregulation of several proinflammatory processes at day 2, and a commonality in upregulated inflammatory response proteins at both day 2 and day 28, indicting ongoing inflammation. Infarct [^18^F]LW223 uptake at day 2 correlated with infarct size (*p = *0.0016, R^2^ = 0.73) and cardiac dysfunction at day 28 (*p = *0.0020, R^2^ = 0.82).

**Conclusion:**

[^18^F]LW223 identifies a persistent and predominantly macrophage-driven inflammatory response with early [^18^F]LW223 infarct binding associated with later cardiac dysfunction.

**Supplementary Information:**

The online version contains supplementary material available at 10.1007/s00259-025-07691-4.

## Introduction

Myocardial infarction (MI) remains one of the leading causes of death worldwide, with a global prevalence of 3.8% in people under sixty years old, rising to 9.5% in those over sixty [1]. Heart failure as a consequence of MI, is also increasing due to improved survival [2]. The early inflammatory response following MI is thought to be a key determinant of late adverse ventricular remodelling and heart failure [3]. The ability to measure and to track the inflammatory response in the heart, using non-invasive imaging such as positron emission tomography (PET), is key to the development of novel therapeutic approaches [4]. Such an approach would allow patient stratification in order to identify those who are most likely to benefit from novel anti-inflammatory therapies in the setting of acute MI.

The 18 kDa translocator protein (TSPO) is a mitochondrial protein involved in the transport of cholesterol for steroidogenesis, in addition to other mitochondrial processes [5]. It has been an imaging target for the investigation of inflammation as it is highly expressed by activated proinflammatory cells of the immune system, particularly microglia in the brain [6] and M1-like macrophages in the periphery [7]. We previously developed the TSPO radiotracer [^18^F]LW223, which is the first fluorinated TSPO radiotracer with target binding independent of the rs6971 human genetic polymorphism [8]; resolving a major bottleneck to widespread use of TSPO PET imaging in the clinic. The rs6971 genetic polymorphism changes the way in which previously developed ligands bind to TSPO, resulting in a trimodal binding distribution pattern [9]. This means that the majority of previously developed TSPO ligands require genetic pre-screening and complex corrections in order to account for the differences in TSPO binding levels at baseline caused by rs6971. For certain ligands it totally precludes their use in the genotype known as low affinity binders [10, 11]. We have since demonstrated that [^18^F]LW223 has good in vivo characteristics, such as low non-displaceable binding, which enables high sensitivity imaging, and allows the use of simplified outcome measures in place of complex kinetic modelling outputs [12, 13]. Clinical translation of [^18^F]LW223 is now underway [14].

Using [^18^F]LW223, we have previously demonstrated an increased macrophage-driven TSPO signal at 7 days following MI in a rat permanent coronary artery ligation model, using a novel analysis approach that removed the need for an additional perfusion scan [8]. Previous studies, with the polymorphism affected TSPO radiotracer [^18^F]GE180 [15], demonstrated elevated TSPO levels in the myocardium within the first week of MI in both mice and humans. Additionally, there was evidence of further increases in the TSPO PET signal at later stages during heart failure following permanent ligation in mice [16–18]. The temporal pattern of TSPO expression in the myocardium following MI has not been described beyond the first week following ischemia with reperfusion injury, an MI model relevant to patients who have received successful percutaneous coronary intervention [19]. Additionally, the cells and proteins driving observed changes in TSPO during cardiac reperfusion injury are unknown. Studies are needed to investigate the utility of the improved TSPO PET radiotracer [^18^F]LW223 in mapping longitudinal inflammation, and assess whether [^18^F]LW223 has prognostic value as an early imaging biomarker with future potential to identify patients for anti-inflammatory therapies post-MI.

This study set out to; 1) map the spatial and temporal expression of TSPO in a clinically relevant rat reperfusion model of MI using a longitudinal study design, 2) phenotype the TSPO positive cell and proteome profiles underpinning the pattern of TSPO expression measured by [^18^F]LW223 PET and 3) establish whether there is an association with the observed [^18^F]LW223 signals and cardiac dysfunction. We hypothesised that the [^18^F]LW223 signal will reflect the TSPO response overtime, and that this response will be representative of CD68^+^ inflammatory cell activity. In addition, we hypothesise that the early [^18^F]LW223 signal, which is reflective of the CD68^+^ macrophage response, will be associated with later cardiac dysfunction.

## Methods

### Rat myocardial infarction reperfusion injury model

All experiments were authorised by the local University of Edinburgh Animal Welfare and Ethical Review Committee and in accordance with the Home Office Animals (Scientific Procedures) Act of 1986. Male Sprague–Dawley rats were utilised in this study, 11 rats in the longitudinal in vivo PET/CT imaging cohort (5 MI, 6 sham) and 32 rats in the time-point matched tissue imaging cohort (12 MI, 12 sham and 8 naïve) which together with the day 28 tissue from the longitudinal experiments covered all imaging time-points. For the baseline naïve cohort (day 2), 10 rats were imaged. All rats were approximately 6–7 weeks old and 202 ± 5 g (mean ± SEM, range 151‒251 g) at the beginning of the study (day 0). Full details of the myocardial infarction with reperfusion surgical procedure can be found in the supplementary.

### Precursor synthesis

The chloride precursor of LW223, (*R*)−3-chloromethyl-(*N*-*sec*-butyl)-*N*-methyl-4-phenylquinoline-2-carboxamide was prepared in four steps from known intermediate, 3-methyl-4-phenylquinoline-2-carboxylic acid. This was conducted as previously described [8].

### Radiotracer production

[^18^F]LW223 was produced as previously described [8]. Briefly, a nucleophilic substitution of the chloride precursor material with [^18^F]fluoride on a TRACERlab FX_FN_ module produces a crude product, which is purified by semi-preparative chromatography and reformulated to yield [^18^F]LW223 in 10% ethanol/saline. Radiochemical yield [20] is consistently > 50%, with molar activity in excess of 100 GBq µmol^−1^. The radiochemical purity in all productions of the tracer was > 99%, as observed by analytical HPLC.

### PET/CT imaging

All PET/CT imaging was carried out in the same individual rats longitudinally (sham and MI), with the exception of the naïve cohort which were scanned at day 0. On the day of the scan, anaesthesia was induced and maintained with 1.5%–2.5% isoflurane (50:50 oxygen/nitrous oxide, 1 l/min) and an intravenous line was established in the tail vein for radiotracer administration. [^18^F]LW223 was delivered by intravenous bolus injection, 23.2 ± 1.4 MBq (mean ± SEM, range 4.6‒60.5 MBq). Immediately prior to CT, a CT contrast agent (250 µl Fenestra + 50 µl Mvivo Au, Medilumine Inc, Canada) was administered by intravenous bolus injection through the tail vein.

Data were acquired using a PET/CT small animal scanner (nanoPET/CT, Mediso, Hungary) over 100 min, beginning immediately following radiotracer injection. Further details on the imaging and reconstruction parameters can be found in the supplementary.

### Kinetic modelling

Reconstructed scans were imported into PMOD version 4.2 (PMOD Technologies, Switzerland). Two-tissue compartment modelling was performed in the PKIN module using an image derived input function sampled from the aorta which was then corrected using a population based radiometabolite curve, a whole blood to plasma ratio of 1.84 [8, 12] and a delay of 30 s. Details of the kinetic modelling process, and outcome parameters, are shown in the supplementary methods and Supplementary Table 1.

### Cardiac polar plot analysis of [^18^F]LW223 binding

**Fig. 1:**
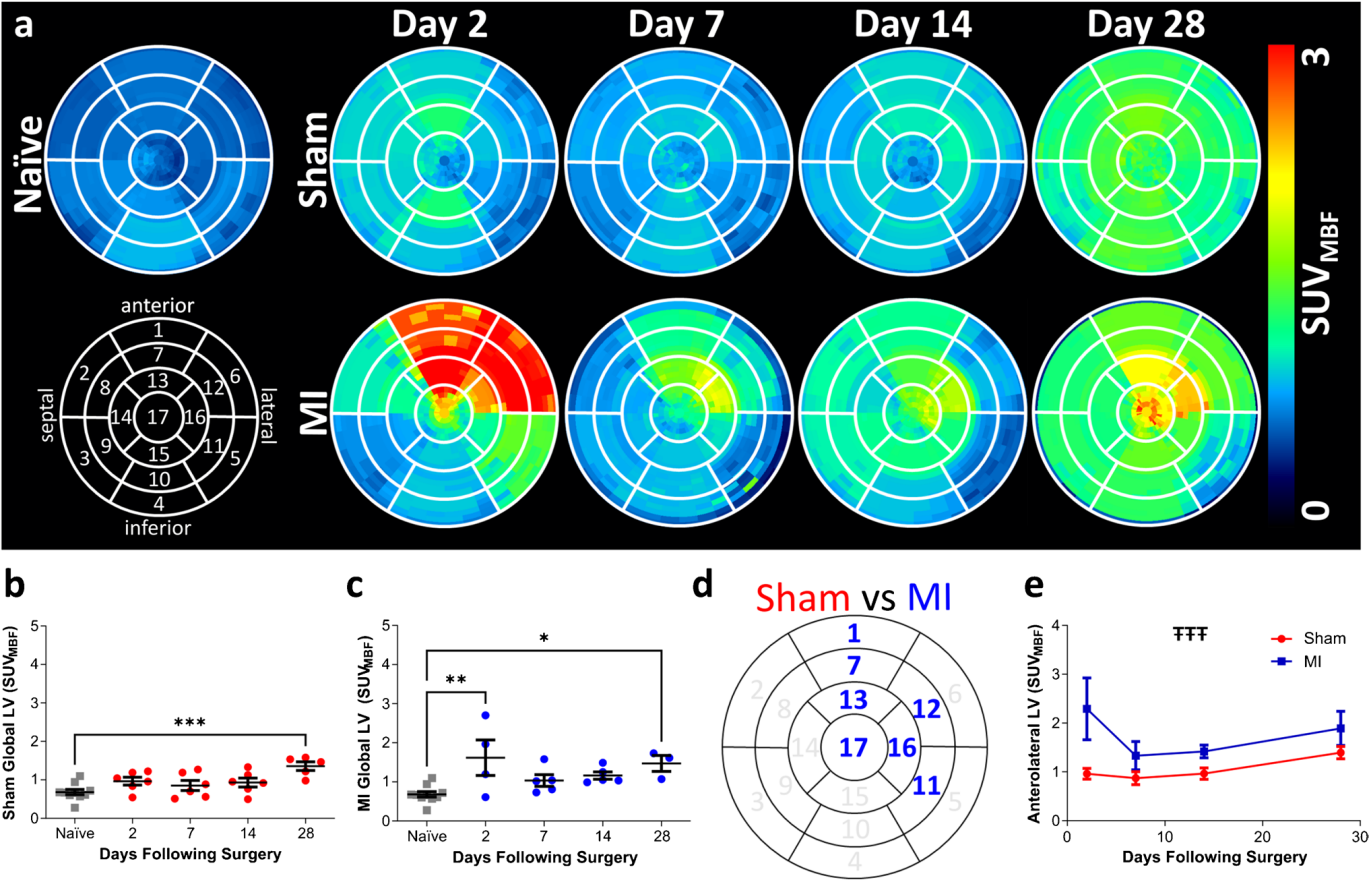
Longitudinal Cardiac PET with [^18^F]LW223 shows a high TSPO signal within the infarct of the MI cohort, peaking at day 2. **a**) Longitudinal cardiac polar plots averaged at each time-point within the sham and cohort. The infarcts within the MI cohort were located within the anterolateral wall. The averaged naïve example is a single time-point only (day 0). **b**) Quantification of [^18^F]LW223 signal across the whole left ventricle for the sham vs. naive cohort (equivalent to day 0). **c**) Quantification of [^18^F]LW223 signal across the whole left ventricle for the MI vs. naïve cohort. **d**) Summary polar plot diagram, highlighting in blue, segments which are statistically different (increased) in the MI cohort vs. sham. **e**) The average [^18^F]LW223 signal across the segments highlighted in panel D. All results are shown as the mean or mean ± SEM. For all panels, naïve *n = *10, sham day 2 *n = *6, sham day 7 *n = *6, sham day 14 *n = *6, sham day 28 *n = *5, MI day 2 *n = *4, MI day 7 *n = *5, MI day 14 *n = *5, MI day 28 *n = *3. * indicates *p ≤ *0.05, ** indicates *p ≤ *0.01, *** indicates *p ≤ *0.001 and **** indicates *p ≤ *0.0001 for one-way ANOVA with post-hoc Dunnett’s vs. the naïve cohort. ŦŦŦ indicates *p ≤ *0.001 using two-way ANOVA for Sham vs. MI

**Fig. 2:**
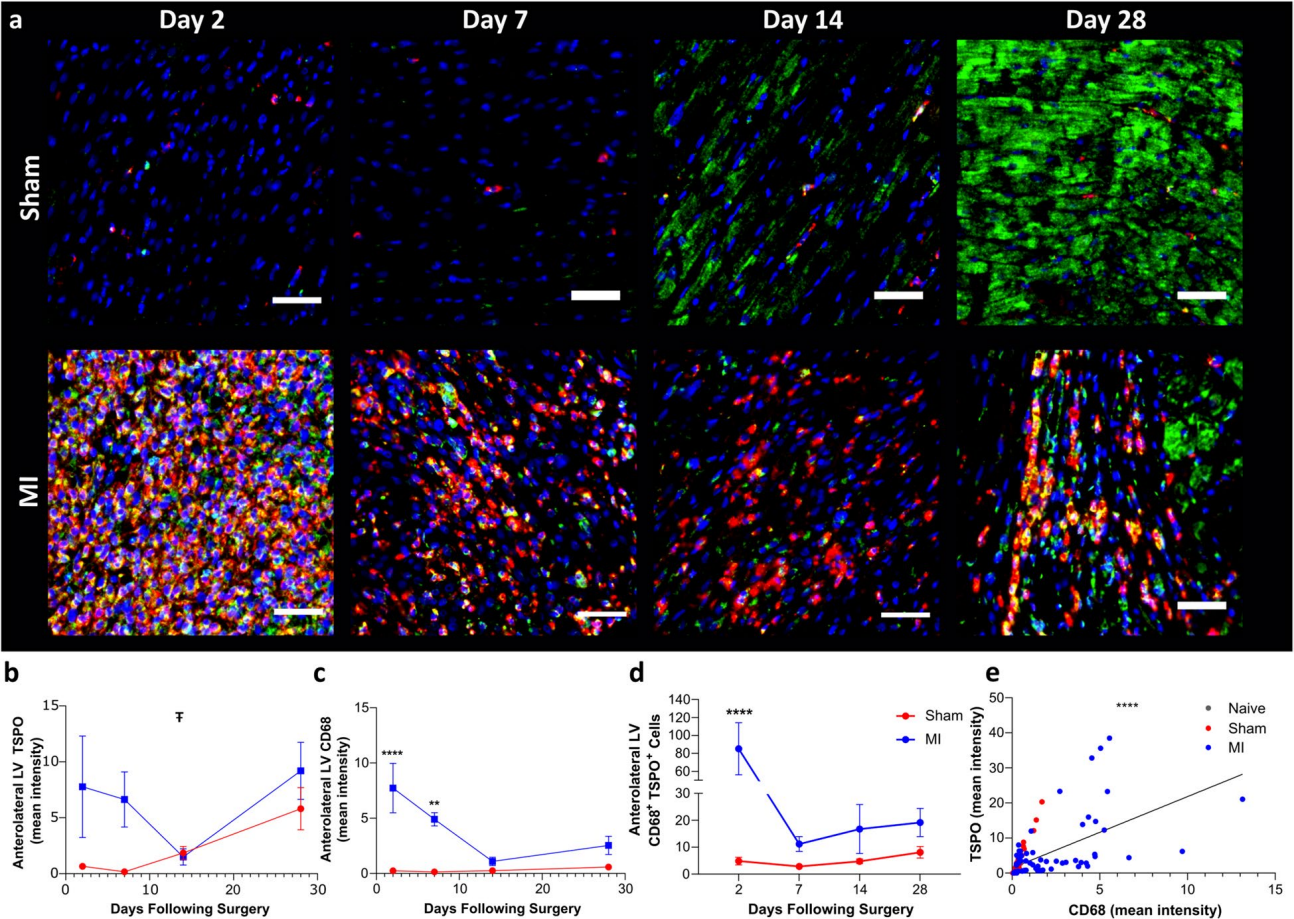
Histological evaluation of cardiac tissue from sham and MI cohorts. **a**) Immunofluorescence staining within the anterolateral wall showing CD68 (red), TSPO (green) and DAPI nuclear counterstain (blue), which is the site of the infarct in animals with MI. Scale bars = 50 µm. **b**) Mean TSPO immunofluorescent staining signal intensity and comparison between sham and MI cohorts at the different time-points, with **c)** equivalent CD68 immunofluorescent staining. **d**) Manual counting of TSPO^+^CD68^+^ cells within the anterolateral wall in sham vs. MI cohorts across the different time-points. For panels B–D, sham day 2/day 7/day 14 *n = *4, sham d28 *n = *6, MI day 2/day 7/day 14 *n = *4 and MI day 28 *n = *5. Ŧ indicates *p ≤ *0.05 using two-way ANOVA for sham vs. MI, ** indicates *p ≤ *0.01, *** indicates *p ≤ *0.001 and **** indicates *p ≤ *0.0001 for two-way ANOVA with post-hoc Šídák's between the same time-point in the sham and MI cohort. **e**) Comparison of the mean TSPO and CD68 immunofluorescent staining intensities in naïve, sham and MI cohorts across all ROI’s (Global ventricle, anterolateral/infarct, infarct border, remote inferoseptal) and time-points (day 2 naïve/sham/MI, day 7, day 14, day 28 sham/MI) measured

Using [^18^F]LW223, and other TSPO radiotracers, for PET imaging within the heart requires perfusion correction, particularly following myocardial infarction with and without ligation reperfusion [8, 16]. In this study, a standardised uptake value (SUV) corrected for myocardial blood flow (MBF) was used. The analysis pipeline for SUV_MBF_ calculation is shown in Sup.Fig. 1. The polar plots shown within this manuscript are a visual average of the individual rats within the specified groups. There were six animals in the longitudinal sham cohort, within which one scan at the day 28 timepoint which was not usable due to technical issues. There were five animals in the longitudinal MI cohort, within which one scan was not usable at the day 2 timepoint and two scans were not usable at the day 28 timepoint, again due to technical difficulties. Sup.Fig. 2 shows that, while systematically biased, MBF assessed by de Grado modelling positively correlates (*p ≤ *0.0001) with the 2TCM outcome measure *K*_*1*_, which for [^18^F]LW223 in the heart is a proxy measure of perfusion [8] (Sup.Fig. 2.a). When comparing *SUV*_*MBF*_ with *BP*_*TC*_, there is a positive correlation (*p ≤ *0.0001, Sup.Fig. 2.b).

### Tissue processing and histology

Tissue was fixed in 10% neutral buffered formalin for 48‒72 h, wax processed and sectioned at 5 µm. To visualise cellular and tissue structure, H&E staining was performed as stated within the supplementary. The CD68 (a macrophage marker) and TSPO stain was carried out using a double tyramide signal amplification (TSA) visualisation, as described previously [21] using the protocol optimised in our previous manuscript [8]. Full details of the immunofluorescence straining, imaging and quantification can be found within the supplementary.

### Cardiac troponin i measurement

At 24 h following surgery, heparinised blood samples (< 500 µl) were collected from the tail vein and centrifuged at 2000 × g for 3 min. The plasma was then collected and diluted in deionised water (1:5 dilution) before being analysed for cardiac troponin I (ARCHITECT_*STAT*_ hs-Tnl assay, Abbott Laboratories, USA) on the Abbott ARCHITECT ci4100 analyser. Troponin I values greater than 2,500 ng/L were classified as an MI, with measurements ranging between 2,583 and 10,970 ng/L in the MI cohort and between 16 and 1,504 ng/L in the sham cohort.

### Echocardiography

At 28 days following the surgical procedure, echocardiography was performed using a VisualSonics Vevo 3100 ultrasound imaging system (FUJIFILM VisualSonics, Canada) to measure ejection fraction, fractional shortening and fraction area change. Details can be found in the supplementary.

### Proteomics

Proteomics was carried out in pooled cardiac tissue, with 4–6 mid-hearts used for each time-point. For the infarcted tissue sampling, both infarct and the immediate penumbra were included. Details of the proteomic experimental procedure and analysis can be found in the supplementary.

### Statistical analysis

Other than proteomics, all statistical analysis was performed using Prism version 9 (GraphPad, USA). All graphical results are displayed as the mean ± standard error of the mean (SEM). Normality analysis was based on D'Agostino-Pearson testing, and the following statistical tests were used as indicated in the figure legends; one-way ANOVA with post-hoc Dunnett’s, Kruskal–Wallis test with a post-hoc Dunn’s, two-way ANOVA with and without post-hoc Šídák's, Pearson correlation and unpaired t-test. Where there is ANOVA level significance, a post-hoc comparison test was conducted.

## Results

### TSPO expression is highest within the infarcted myocardium, peaking at day 2 and remaining elevated throughout the study

**Fig. 3:**
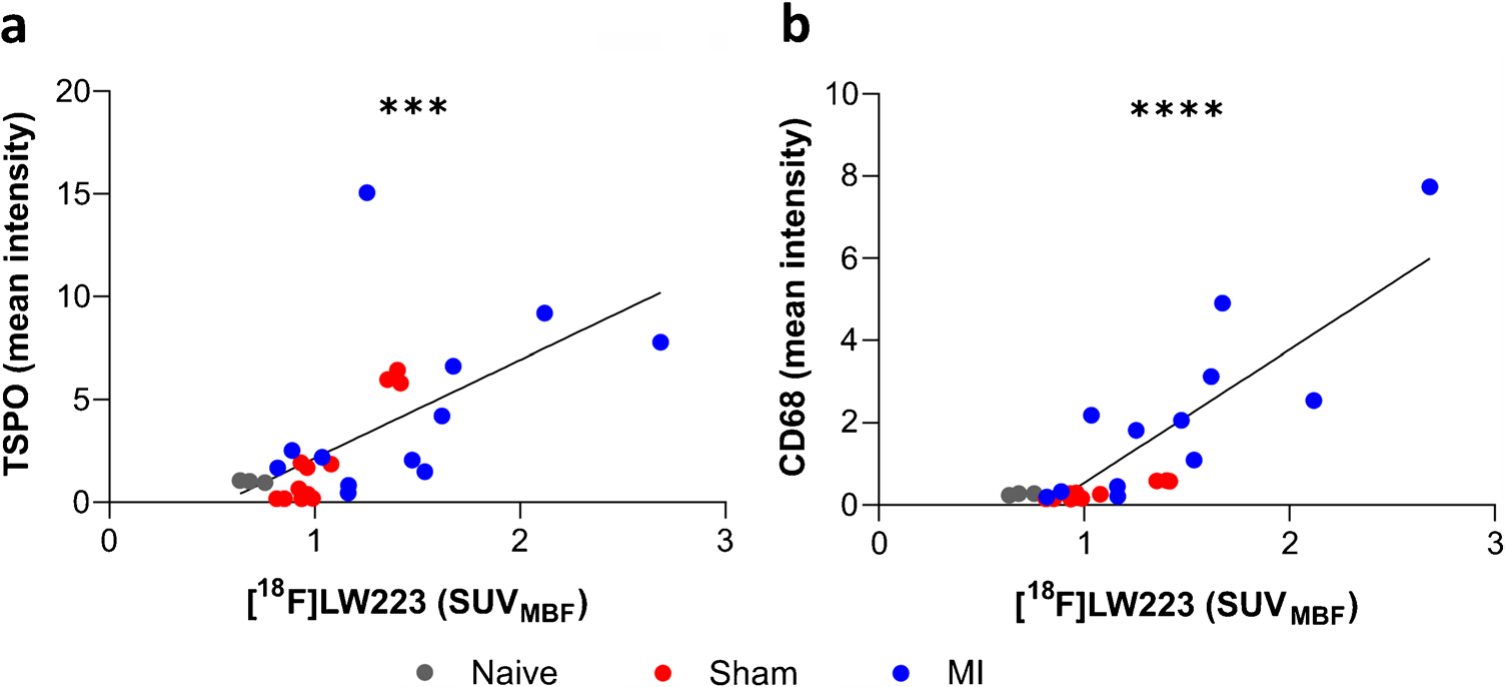
TSPO and CD68 histology quantification validates the in vivo [^18^F]LW223 PET findings. **a**) Comparison of the mean TSPO immunofluorescent staining intensity averaged across each time-point in all global, Anterolateral/infarct and inferoseptal ROIs measured in naïve, sham and MI cohorts vs. *in* vivo PET [^18^F]LW223 signal in VOI/segments (Global heart, segment 12 and segment 9) which are equivalent to histology ROIs. **b**) Comparison of the mean CD68 immunofluorescent staining vs. *in* vivo PET [^18^F]LW223 signal as it was performed for TSPO. *** indicates *p ≤ *0.001 and **** indicates *p ≤ *0.0001 for Pearson correlation

**Fig. 4:**
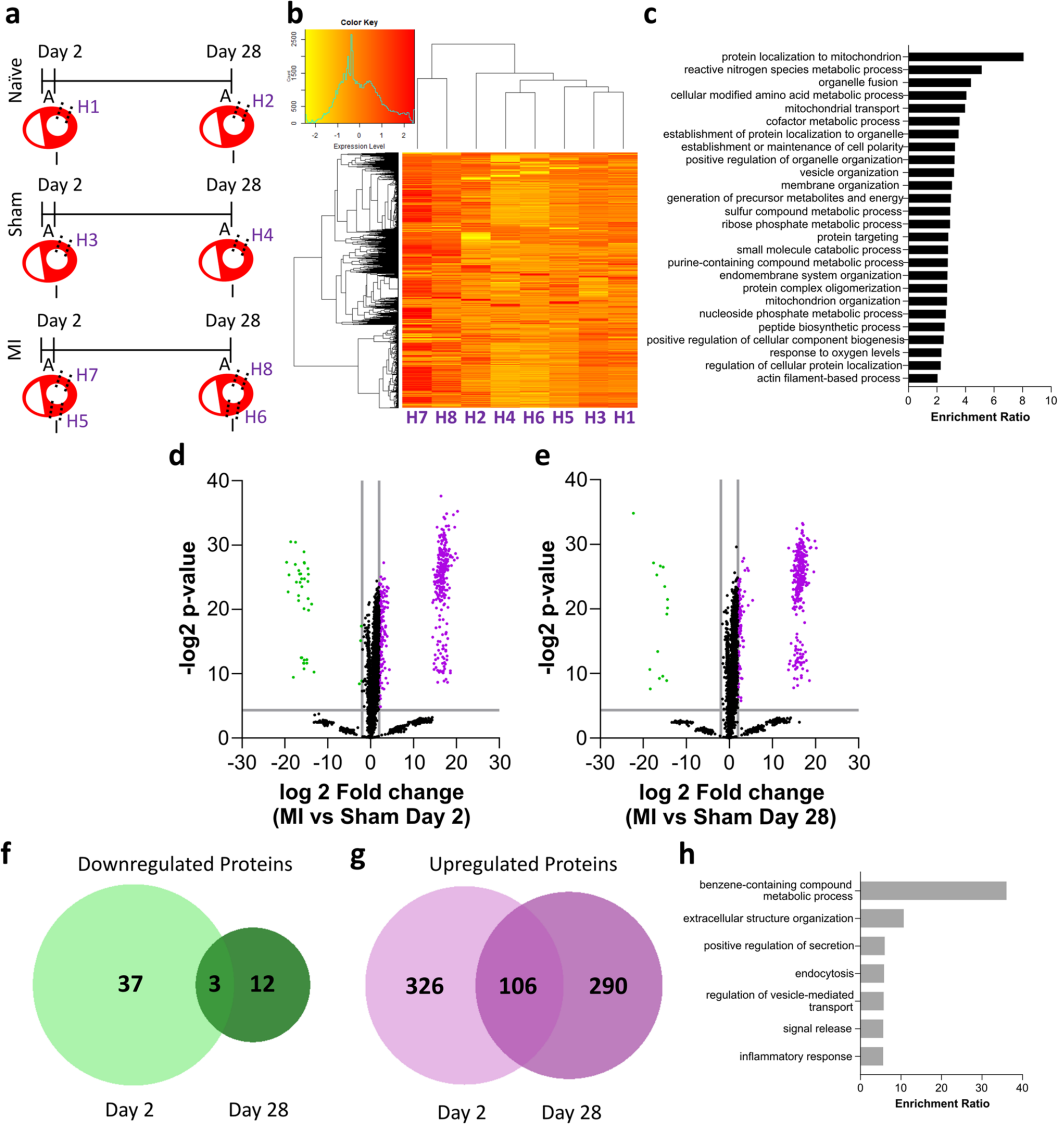
Cardiac proteomic analysis demonstrates an infarct driven proteomic profile, with a TSPO cluster associated with mitochondrial processes, and an upregulation of overlapping inflammatory proteins at day 2 and 28. **a**) Schematic showing the time-points and location of the sampled cardiac tissue, A = anterior and I = inferior. **b**) Whole proteome heatmap across different tissue groups showing a unique infarct driven signature in groups H7 and H8. **c**) TSPO protein cluster GO Terms and their enrichment, generated from the row dendrogram in panel B. **d**) Volcano plot showing significantly (− log2 *p* > 4.3219) upregulated (log2 fold change > 2) and downregulated (log2 fold change > − 2) proteins in day 2 MI anterolateral tissue vs. day 2 sham anterolateral tissue. **e**) The same volcano plot shown for day 28 MI anterolateral tissue vs. day 28 sham anterolateral tissue. **f**) Venn diagram of significantly downregulated proteins at both day 2 and day 28, showing minimal overlap. **g**) Venn diagram of significantly upregulated proteins at both day 2 and day 28, showing an overlap of 106 proteins. **h**) GO terms summarising the overlapping upregulated proteins

**Fig. 5:**
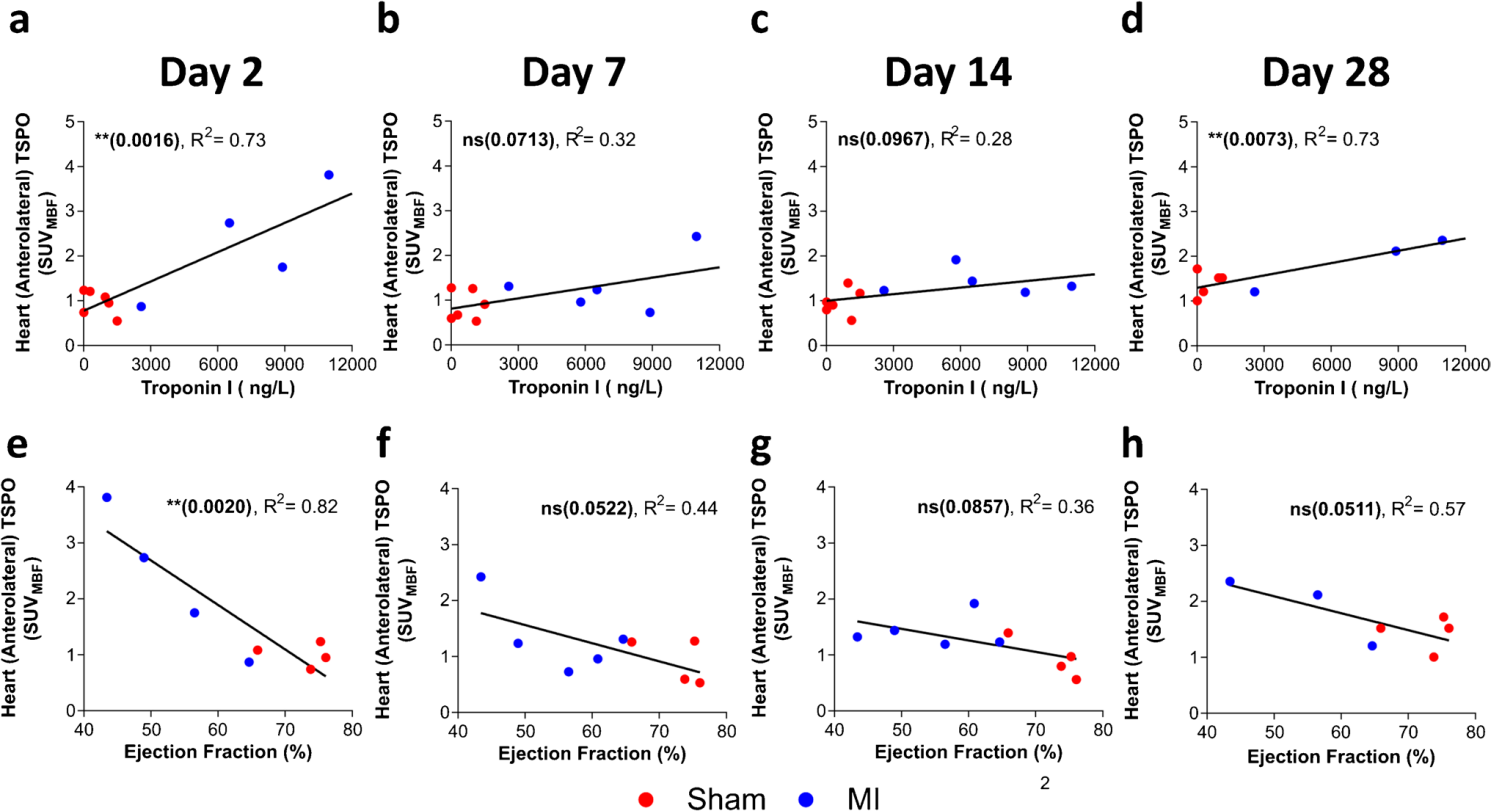
[^18^F]LW223 signal within the infarct/anterolateral wall correlates with infarct severity and predicts later systolic dysfunction. **a**) Comparison of plasma cardiac troponin I levels measured 24 h after surgery and the [^18^F]LW223 signal within the infarct/anterolateral wall at day 2, **b**) day 7, **c**) day 14 and **d**) day 28. Sham *n = *5–6, MI *n = *3–5. **e**) Comparison of ejection fraction assessed by ultrasound and the [^18^F]LW223 signal within the infarct/anterolateral wall at day 2, **f**) day 7, **g**) day 14 and **h**) day 28. Sham *n = *4, MI *n = *3–5. For all panels, * indicates *p ≤ *0.05, ** indicates *p ≤ *0.01 and ns indicates *p* > 0.05 for Pearson correlation

Average polar plots for all cohorts and time-points are shown in Fig. 1.a. Within the shams at day 2, a small increase in [^18^F]LW223 binding is evident, along with a general increase in [^18^F]LW223 binding across the whole left ventricle at day 28 (Fig. 1.a&b, Sup.Fig. 3). Within the MI cohort, [^18^F]LW223 binding is highest at day 2 in the infarcted region of the anterolateral wall before quickly decreasing within the first week (Fig. 1.a&c, Sup.Fig. 4). In some segments of the infarcted region, [^18^F]LW223 binding remains elevated throughout the study (e.g. Segment 7, Sup.Fig. 4.c). In addition, segments with high signal at day 2 also displayed a high signal at day 28. (Sup.Fig. 5). As well as an infarct specific pattern, the same general increase in [^18^F]LW223 binding observed within the sham cohort across the whole ventricle is also evident within the MI cohort towards day 28 (e.g. Segment 9, Sup.Fig. 4.j). Overall, the highest [^18^F]LW223 binding is present within the infarcted anterolateral wall of the MI cohort across the study (*p ≤ *0.001 for sham vs. MI, Fig. 1.d&e, Sup.Fig. 6.a, c, e, g, o, p & q), with no differences between the sham and MI observed in the remote inferoseptal regions (e.g. Sup.Fig. 6.d, j & k). To compare the TSPO response observed in this reperfusion model with the permanent ligation model, we reanalysed our previously published data using permanent ligation [8] to calculate the SUV_MBF_. Comparison of the TSPO response at day 7 in both models show a more intense signal in the permanent ligation model (Sup.Fig. 7).

In order to validate our longitudinal in vivo PET findings shown in Fig. 1, additional rats were generated for each cohort corresponding to the imaging time-points (Fig. 2, Sup.Fig. 8). H&E staining was used to assess tissue morphology, confirm infarct location, and guide ROI placement for immunofluorescent analysis (Sup.Fig. 8.a). A TSPO and CD68 double stain was performed to assess TSPO expression both within and outside monocyte-lineage cells, such as macrophages, across all cohorts and time-points (Fig. 2.a, Sup.Fig. 8.b). The individual channels from the examples shown in Fig. 2.a are shown in Sup.Fig. 9, and examples of antibody controls are provided in Sup.Fig. 10. The same patterns observed within the in vivo PET dataset are present within the ex vivo tissue imaging. TSPO and CD68 signal intensity, and manually quantified TSPO^+^CD68^+^ cells, are highest within the infarct of the MI cohort at day 2, and this inflammatory response never fully recovers to sham levels (Fig. 2.b-d). In addition, a general TSPO increase across the ventricle was observed for both sham and MI cohorts up to 28 days post-infarction, with a prominent increase in cardiomyocyte TSPO staining evident (Fig. 2.a & Sup.Fig. 8.b, day 14 & 28 panels). The staining patterns in Fig. 2 and Sup.Fig. 8 indicate an age-related increase in cardiomyocyte TSPO expression in addition to the inflammatory TSPO signal. This increase in cardiomyocyte TSPO was also evident in histology performed in aged matched (day 2 and day 28) naïve animals, confirming that the effect is age-related rather than due to either the sham or MI procedure (Sup.Fig. 11). Sup.Fig. 12 shows the changes in % TSPO^+^CD68^+^ cells in relation to the total TSPO^+^ cells across the different cohorts and time-points. At day 2 within the infarcted region of the MI cohort, 55.2% (mean of 85 cells across measured grids) of the TSPO + cells are TSPO^+^CD68^+^ vs. only 11.9% (mean of 5 cells across measured grids) within the sham anterolateral wall (Sup.Fig. 12.c&g). Infarct TSPO^+^CD68^+^ cells decrease to 23.6% of the total TSPO^+^ cells (mean of 21 cells across measured grids) as cardiomyocyte TSPO expression increases towards day 28 (Sup.Fig. 12.j). Across all cohorts, regions and time-points, TSPO expression positively correlated with CD68 expression (*p ≤ *0.0001, Fig. 2.e). The average TSPO and CD68 expression across each time-point for the different regions and cohorts positively correlated with [^18^F]LW223 binding (Fig. 3), indicating that our imaging approach was accurate for the target (TSPO) and cell type of interest (macrophage).

### Proteomic analysis reveals an upregulation of overlapping inflammatory proteins at each TSPO expression peak, indicating an ongoing inflammatory process

To further characterise the TSPO response in the heart following myocardial infarction, tissues were analysed using proteomics at the peak in [^18^F]LW223 binding at day 2, and at day 28. Aged-matched naïve and sham cohorts were used as controls, in addition to non-infarcted remote myocardium as shown in Fig. 4.a. Hierarchical clustering of the proteome across all tissue groups demonstrated that infarcted tissue (both infarct and immediate penumbra), at both day 2 and day 28, share a distinct proteome clustering relative to all the other groups (Fig. 4.b). Extraction of the TSPO cluster from this heatmap demonstrates that the main processes associated with this protein are related to mitochondrial function and metabolism in the myocardium (Fig. 4.c). To characterise the proteome at the day 2 TSPO peak, upregulated (fold change > 4, *p* < 0.05, purple) and downregulated (fold change < − 4, *p* < 0.05, green) proteins between day 2 sham and day 2 MI infarct tissues were investigated (Fig. 4.d). Amongst the varied GO terms returned for the upregulated proteins, there were multiple terms associated with the innate immune system and tissue response to injury (Sup.Fig. 13.a). Characterisation of the proteome at the study endpoint (day 28) demonstrated mainly processes involving tissue remodelling (Sup.Fig. 13.b). There were no GO terms associated with the down-regulated proteins (Fig. 4.d&e). Overlapping hits of downregulated and upregulated proteins across the day 2 and day 28 time-points (Fig. 4.f and Fig. 4.g respectively), revealed few overlapping downregulated proteins (3) but a considerable overlap in upregulated proteins (106). Among the GO terms returned for the upregulated overlapping proteins was a group of “inflammatory response” proteins. This indicates commonality in immune cell activity across the day 2 and day 28 time-points, suggesting an ongoing inflammatory response and support the findings from our PET and histology datasets.

### Cardiac [^18^F]LW223 PET at day 2 post-infarction correlates with infarct size and systolic dysfunction at day 28 post-injury

Troponin I measurements correlated with the [^18^F]LW223 PET signal at day 2 (*p = *0.0016) and day 28 (*p = *0.0073), indicating larger infarcts resulted in a higher cardiac inflammatory response (Fig. 5.a-d). Across the MI cohort, systolic dysfunction was evident by cardiac ultrasound at day 28 (Sup.Fig. 14), with the extent of dysfunction correlating with Troponin I (Sup.Fig. 15). The [^18^F]LW223 PET signal in the infarct and anterolateral wall at day 2 correlated with ejection fraction measured at day 28 (r^2^ = 0.82, *p = *0.0020, Fig. 5.e). The anterolateral wall [^18^F]LW223 signal at day 2 also correlated with other measures of systolic dysfunction measured at day 28, specifically fractional shortening (r^2^ = 0.76, *p = *0.0050, Sup.Fig. 16.a) and fractional area change (r^2^ = 0.57, *p = *0.0303, Sup.Fig. 16.e).

## Discussion

This study set out to assess whether longitudinal [^18^F]LW223 PET could accurately map inflammation, to determine the temporal expression of TSPO in the setting of MI with reperfusion, and to investigate the mechanisms and processes underlying any changes in TSPO expression. We also set out to establish whether early [^18^F]LW223 uptake was associated with cardiac dysfunction. Overall, we demonstrated that TSPO expression peaks early within the infarcted myocardium at day 2, with some regions remaining elevated throughout the remainder of the study and a high day 2 signal being predictive of a high day 28 signal. This pattern is caused by localised infiltration of TSPO^+^CD68^+^ macrophages and is associated with an upregulation of a set of inflammatory proteins present at both day 2 and day 28. This early inflammatory signal correlated with the later development of cardiac dysfunction.

Our findings confirm previous studies demonstrating a TSPO response associated with both changes in inflammatory cells and cardiomyocytes. Using another TSPO radiotracer ([^18^F]GE180) in a mouse permanent ligation MI model, TSPO PET imaging performed at 1, 4 and 8 weeks following MI revealed a peak at 1 week, driven by CD68^+^ cells, and a later peak at 8 weeks, driven by cardiomyocytes [16]. While a direct comparison with our study is limited by the difference in species (mice vs. rat), and injury approach (permanent ligation vs. reperfusion), their findings of an early inflammatory cell driven TSPO signal and a later cardiomyocyte associated TSPO signal, are partially in agreement with the outcomes of our study. However, our study also demonstrates a persistent and ongoing inflammation-driven TSPO response which never fully returned to the levels observed in our age matched sham controls. Given that the extent of this persistent day 28 inflammation is higher in segments which had a high day 2 inflammatory signal, and the association between day 2 TSPO and later cardiac dysfunction, overall, our results indicate that more severe inflammation causes increased adverse ventricular remodelling. In addition, comparison of our data in the reperfusion model with our previously published data using the permeant ligation model indicates that the TSPO response observed with permanent ligation is greater at day 7. However, in mice it is known that peak macrophage activity differs between these injury approaches [22]. Therefore, in the absence of longitudinal data with [^18^F]LW223 in permanent ligation setting, these results should be interpreted with caution.

The level of the observed age-related increase in cardiomyocyte TSPO expression should be viewed in the context of the relatively young age of the rats at the start of our study, and therefore the rapid period of growth which they undergo, increasing from ~ 200 g to > 300 g by day 28., Our study is not the first to show that TSPO expression changes with age. In humans, it known that neuronal TSPO increases with age [23]. However, it is not yet known if a similar age-related change occurs in human hearts, and therefore, this aspect warrants further investigation and highlights the importance of controlling for age in future studies.

The link between a prolonged or elevated inflammatory response in the acute phase following MI, and subsequent development of adverse ventricular remodelling leading to a higher risk of heart failure is well established [3]. For the first time, our study establishes the association between early cardiac [^18^F]LW223 PET imaging of the acute cellular inflammatory response and later systolic dysfunction at day 28. This association warrants future investigation and suggests the prognostic potential of cardiac [^18^F]LW223 PET imaging for LV dysfunction following MI. Such prognosis is one of the key goals of any inflammatory imaging approach in the context of MI, and is vital for patient stratification and the development of anti-inflammatory therapies [24]. For example, TSPO PET imaging could be used to identify individuals who are most likely to benefit from novel anti-inflammatory therapies, removing unnecessary exposure to increase infection risk and reducing the high financial burden from these approaches, such as anti-interleukin-1β therapy with canakinumab [25]. Other imaging options explored in the context of acute inflammation following MI, such as the radiotracer [^18^F]FDG (fluorodeoxyglucose), have also been shown to associate with left ventricular impairment [26]. However, use of [^18^F]FDG requires complex patient preparation to supress the high cardiomyocyte glucose uptake, and TSPO remains one of the most specific imaging targets for assessing pro-inflammatory macrophages [7]. In addition, we demonstrated that infarct size, assessed by cardiac troponin, was closely associated with both early and late [^18^F]LW223 binding within the infarct, indicating that our approach to assess inflammation is proportional to the level of myocardial injury.

As previously established, perfusion correction of TSPO imaging within the infarcted myocardium is essential [8, 12, 16]. In this study, we have validated the use of a new translatable outcome measure, termed *SUV*_*MBF*_. This new approach is a simplification of our original study using invasive blood sampling to calculate the binding potential, corrected for transfer kinetics (*BP*_*TC*_) [8], and is justified by our data showing that [^18^F]LW223 has a low non-displaceable volume [13]. Removing the need to sample radioactivity in the blood makes this new approach more translatable. In addition, acquisition of only the first four minutes of the scan, and an average image at binding equilibrium, is required, further increasing the translatability compared with our previously established full kinetic modelling approach. Given the kinetics of [^18^F]LW223 in the heart, and stability of SUV from 20 min onwards [8, 12], future clinical imaging protocols could enable calculation of *SUV*_*MBF*_ using a 30 min scan, dependant on when binding reaches equilibrium. Since the first publication of [^18^F]LW223 in 2021, the potential of this approach in the context of cardiology has been widely recognised and discussed [4, 27–29]. [^18^F]LW223 has moved along the translational pipeline and has now been assessed in non-human primates, and a small number of healthy human volunteers [14]. The results from these advanced studies, as well as our follow-up preclinical investigations [13, 30], confirm what we have previously demonstrated in terms of radiotracer specificity, sensitivity, metabolism and kinetics. [^18^F]LW223 remains an excellent candidate for further clinical translation for cardiological indications and beyond.

While TSPO appears to be the most relevant current target for the assessment of pro-inflammatory macrophages [7], other inflammatory targets and radiotracers are also available, such as CXCR4 [31]. The combination of multiple radiotracers targeting different inflammatory aspects within the same individual holds potential for further phenotyping of the immune response, as discussed in a recent review [32].

The main limitation in our study is the nature of our preclinical model. While the rat model of MI utilised is typical of the field, the age of these rats are equivalent to adolescent humans who do not typically experience MI. Also, this model does not assess the impact of common co-morbidities, such as diabetes, which are known to influence the innate immune response in this context [33]. Additionally, while our results were appropriately powered and statistically significant, the number of final datapoints in the infarcted cohort was lower than anticipated.

## Conclusion

This study demonstrates that, following MI, there is an acute cellular inflammatory response within the infarcted myocardium which persists for several weeks. The acute phase of this macrophage-driven response, measured using [^18^F]LW223 PET, is associated with the extent of myocardial injury and later systolic dysfunction. This non-invasive approach to the imaging and quantifying the macrophage-driven response following MI, using outcome measures amenable to clinical translation, holds promise for future application in the clinical cardiology setting.

## Supplementary Information

Below is the link to the electronic supplementary material.

Supplementary file1 (DOCX 187697 KB)

## Data Availability

The datasets generated during and/or analysed during the current study are available from the corresponding author on reasonable request.
