# Supplementary material for: Longitudinal [18F]LW223 PET imaging of macrophage-driven inflammation following myocardial infarction in a rat model: implications for left ventricular remodelling
Source: Eur J Nucl Med Mol Imaging. 2025 Dec 12;53(5):3340–50. doi: 10.1007/s00259-025-07691-4 (PMC13013395; doi:10.1007/s00259-025-07691-4)
Supplement: Supplementary file 1 — Supplementary file1 (DOCX 187697 KB) [file 259_2025_7691_MOESM1_ESM.docx]

**Supplemental**

**Title:**

Longitudinal [^18^F]LW223 PET imaging of macrophage-driven inflammation following myocardial infarction in a rat model: implications for left ventricular remodelling

**Authors:**

Mark G. MacAskill^1,2^, Victoria J.M. Reid^1,2^, Carlos J. Alcaide-Corral^1,2^, Timaeus E.F. Morgan^1,2^, Lachlan Waddell^3^, Adrian J.W. Thomson^1,2^, Takeshi Fujisawa^1^, Nicholas L. Mills^1^, Judit A. Marti^4^, Dominic Kurian^4^, Thomas M. Wishart^4,5^, Ana Clara Juan De Albuquerque^1^, Ernest Chui^1^, Agne Knyzeliene^1,2^, Viktoria Balogh^1,2^, Catriona Wimberley^2,6,7^, Marc R. Dweck^1^, David E. Newby^1^, Christophe Lucatelli^2^, Sally L. Pimlott^8,9^, Andrew Sutherland^3^, Adriana A.S. Tavares^1,2^*.

**Affiliation:**

^1^Center for Cardiovascular Science, University of Edinburgh, Edinburgh, United Kingdom.

^2^Edinburgh Imaging, University of Edinburgh, Edinburgh, United Kingdom.

^3^School of Chemistry, University of Glasgow, Glasgow, United Kingdom.

^4^The Roslin Institute, University of Edinburgh, Edinburgh, United Kingdom.

^5^Centre for Systems Health and Integrated Metabolic Research, Nottingham Trent University, Nottingham, United Kingdom.

^6^Centre for Clinical Brain Sciences, University of Edinburgh, Edinburgh, United Kingdom.

^7^Institute of Particle and Nuclear Physics, University of Edinburgh, Edinburgh, United Kingdom.

^8^School of Medicine, University of Glasgow, Glasgow, United Kingdom.

^9^NHS Greater Glasgow and Clyde, Glasgow, United Kingdom.

***Corresponding Author:**

Adriana Tavares, Centre for Cardiovascular Science, QMRI, The University of Edinburgh, 47 Little France Crescent, Edinburgh, EH16 4TJ, UK. [adriana.tavares@ed.ac.uk](mailto:adriana.tavares@ed.ac.uk)

**Supplementary Methods:**

**Rat Myocardial Infarction Reperfusion Injury Model**

The animals were housed under standard conditions of 12 h of light and 12 h of darkness, with food and water available ad libitum. For the myocardial infarction procedure, anaesthesia was induced and maintained using isoflurane (0.5%–3% in oxygen, 1 L/min) before buprenorphine (0.05 mg/kg; Alstoe Ltd.) was administered preoperatively for analgesia. Tracheal intubation was achieved under direct vision, and ventilation was maintained with a rodent ventilator according to the manufacturers’ specifications (model 683, Harvard Apparatus, USA). Body temperature was maintained by heated mat and monitored by a rectal thermometer and vital signs, including heart rate and respiration rate, were monitored continuously during the experiments. The skin was incised at the level of the left third and fourth ribs where the pectoral muscles were divided and retracted. Left lateral thoracotomy was then performed. To gain access to the heart, the left lung was gently collapsed by placing a small triangular sponge over the lung. With minimal handling, the pericardium was ruptured and the heart gently exteriorised from the thorax, and a nonabsorbable 4-0 suture was placed around the left anterior descending (LAD) coronary artery just above the bifurcation of the first diagonal and maneuvered back into position. The suture ends were passed through a piece of sterile tubing (~3 mm) before being pulled tight to applying pressure and occlude the LAD. This pressure was maintained using a bulldog clip on the end of the tubing and the area was covered with damp sterile swabs. After 30 mins of occlusion, the pressure on the LAD was removed by opening the bulldog clip to allow reperfusion. The sterile tubing was then removed and the suture around the LAD was left in place. The wound was then closed by interrupted suturing (4-0 non-absorbable suture) of the thoracotomy site before the pectoral muscle were maneuvered back into position and the skin was closed by interrupted subcuticular suturing (3-0 absorbable suture). The rats were given sterile sodium chloride 0.9% 0.01 ml/g fluid therapy subcutaneously and extubated once spontaneous ventilation was established before being housed at 30 °C for 24 hours. An additional dose of Buprenorphine was given the following morning and normal housing conditions were resumed with additional animal monitoring. At the end of the study, animals were euthanised by exposure to carbon dioxide gas in a rising concentration.

**PET/CT imaging**

Immediately following radiotracer administration, a 100-minute emission scan was obtained using 3-dimentional 1:5 mode and re-binned as follows: 18×10 sec; 2×30 sec; 1×60 sec; 2×2 min; 10×5 min; 4×10 min. Two CT scans (semi-circular full trajectory, maximum field of view, 480 projections, 50 kVp, 300 ms and 1:4 binning) were acquired, one for attenuation correction and the other for contrast agent visualisation. PET studies were reconstructed using Mediso’s iterative Tera-Tomo 3D reconstruction algorithm, which includes point spread correction to account for partial volume effects, and the following settings: 4 iterations, 6 subsets, full detector model, low regularisation, spike filter on, voxel size 0.4 mm and 400‒600 keV energy window.

**Kinetic Modelling**

Volumes of interest (VOIs) were manually drawn around the heart using average PET and CT images. A spherical VOI was placed within the aorta using contrast enhanced CT images in order to measure the activity within the blood. To sample the infarct area (or equivalent area in naïve hearts) averaged PET images (0‒100 minutes) were used to place three spherical VOIs at the centre of the infarct territory within the ventricular wall (1.5 mm^3^). The same approach was used to sample the remote region within the mid inferior wall. Kinetic modelling was performed using the two-tissue compartment model (2TCM) to estimate the kinetic rate constants *K_1_* to *k_4_* and *BP_TC_*, with *V_B_* fixed at 0.05. Two-tissue modelling was selected as the preferred model as demonstrated in our previous study [1]. An image derived input function sampled from the aorta was used for this modelling, which was corrected using a population based radiometabolite curve, a whole blood to plasma ratio of 1.84 [1,2] and a delay of 30 seconds. A summary of the kinetic modelling outcome parameters is shown in Supplementary Table 1. Estimates with a standard error greater than 2000% were excluded and the final number of datapoints for each group are also shown in Supplementary Table 1.

**Cardiac Polar Plot Analysis of [^18^F]LW223 binding**

Dynamic PET files were cropped around the heart and imported into the PCARD tool within PMOD version 4.2 (PMOD Technologies, Switzerland). Due to the unique properties of [^18^F]LW223 as discussed in our previous study [1], we were able to utilise the first 4 mins of dynamic PET data to calculate MBF. This was carried out using the de Grado model [3]. It was not possible to accurately estimate the spill-over fraction of blood activity in the left and right ventricle (vLV and vRV) on an individual scan basis. Therefore, an average vLV and vRV of 0.41 and 0.06, respectively, was used across all scans. This was calculated from analysis of the naïve cohort (n=10), where vLV and vRV were left floating. An [^18^F]LW223 mCorr factor of 0.0286 was used based on our previously reported heart radiometabolism data [1]. The resulting 3D segment MBF parameter map was then saved as a DICOM for use in the View tool within PMOD (version 4.2, PMOD Technologies, Switzerland). An *SUV_mean_* heart crop image was created from the frames between 70‒100 min, at which point [^18^F]LW223 binding is at equilibrium during a pseudo-irreversible phase, and imported into the PCARD tool in order to generate a 3D segment *SUV_mean_* parameter map which was also exported as a DICOM. Within the View tool, the image algebra function was used to divide the *SUV_mean_* 3D segment parameter map by the MBF 3D segment parameter map. This perfusion corrected [^18^F]LW223 3D segment parameter map was then imported back into the PCARD tool in order to generate the final *SUV_MBF_* polar plots from which the average *SUV_MBF_* within each segment was exported. The polar plots shown within this manuscript are a visual average of the replicates within those groups. Due to technical issues with radiotracer injections, we were not able to calculate the MBF in 6 scans within the sham cohort (2 at day 7, 4 at day 28). For these animals an average MBF parameter map was created from the other scan timepoints within that same animal and used for *SUV_MBF_* calculation. In the MI cohort we were not able to calculate a MBF in 1 scan which was at the day 7 timepoint. For this incidence, we used an average MBF parameter map created from the MBF at day 2, 14 and 28 to calculate *SUV_MBF_*_._

For validation against our previous analysis approach, which used 2TCM and *BP_TC_* as a way to accurately measure [^18^F]LW223 binding using a single PET scan [1], we calculated BPTC in this current cohort for direct comparison with our SUV_MBF_ data. Sup.Fig.2 shows that, while systematically biased, MBF assessed by de Grado modelling positively correlates (p ≤ 0.0001) with the 2TCM outcome measure *K_1_*, which for [^18^F]LW223 in the heart is a proxy measure of perfusion (Sup.Fig.2.a). When comparing *SUV_MBF_* with *BP_TC_*, there is a positive correlation (p ≤ 0.0001, Sup.Fig.2.b).

**H&E Staining**

Slides were dewaxed through two changes in xylene and rehydrated through graded alcohol before a 5 min incubation in Haematoxylin. The slides were then washed in 1% acid alcohol (5 s) and differentiated in Scott’s tap water substitute (10 s). Slides were then incubated in eosin (5 s) before thoroughly washing and dehydrating through graded alcohol. The slides were cleared in xylene and mounted using synthetic resin.

**CD68 and TSPO** **Immunofluorescence Straining, Imaging and Quantification**

Antigen retrieval was carried out in a pressure cooker using tris-EDTA (pH 9) before an endogenous peroxidase block (3% H_2_O_2_, 15 min) and tissue permeabilisation/ blocking in 10% donkey serum (PBS, 0.1% Triton X-100) for 1 hour room at temperature. The CD68 primary antibody (1/100 in 1% donkey serum, MCA341R, Bio-Rad, USA) was incubated with the slides overnight at 4 °C, followed by incubation with the first secondary antibody (1/750 in 1% donkey serum, donkey anti-mouse-HRP, 715-036-150, Jackson ImmunoResearch, USA) for 1 hour at room temperature. This first secondary antibody was visualised using the TSA system (Opal 540, 1/100 for 4 min, Akoya Biosciences, USA). Microwave antigen retrieval (tris-EDTA pH 9) was then carried out to destroy the CD68-associated antibodies, leaving behind the visualisation product only. An additional endogenous peroxidase and serum block was performed before incubation with the TSPO primary antibody (1/100 in 1% donkey serum, ab109497, Abcam, UK) for 1 hour at room temperature. A subsequent secondary antibody incubation was performed at 1 hour room temperature (1/750 in 1% donkey serum, donkey anti-rabbit-HRP, 711-036-152, Jackson ImmunoResearch, USA) before visualisation with the TSA reagent (Opal 650, 1/100 for 4 min, Akoya Biosciences, USA). The slides were then counterstained with DAPI and mounted. Mouse and rabbit IgG at an equal concentration served as an isotype control. In addition, to ensure there was no crossover of the different tyramide complexes an addition control was performed were the second primary antibody (TSPO) was not added.

Whole tissues were imaged using an Axio Scan.Z1 slide scanner (Zeiss, Germany). Quantification of mean intensity was performed on TIF files using Image J (v1.49, NIH, USA) by drawing regions of interest on H&E sections serial to the immunoassayed slides and applying these regions of interest (ROI) to the immunofluorescent images. In addition, TSPO^+^ cells, TSPO^+^CD68^+^ cells and DAPI stained nuclei were manually counted within the infarct/anterolateral wall. This was performed by applying a coordinated grid system to the ROI and randomly selecting 5 grids in which the cells were manually counted. The grid size was optimised in order to ensure between 100-200 total cells were typically counted per grid.

**Echocardiography**

Anaesthesia was achieved using isofluorane (induction using 4.5% in 100% O2, maintenance at 3%) and the rat was placed supine on a heated monitoring imaging table. All four paws were taped along with Parker Spectra 360 Electrode Gel (Parker Laboratories, USA) to the table ECG electrodes to monitor ECG, respiration and heart rate. Body temperature was measured using a rectal probe and maintained at 37 °C ± 0.5 °C. Thoracic region hair was removed using depilation cream (Nair hair removal cream, Church & Dwight, UK) and Parkers Aquasonic 100 ultrasound gel (Parker Laboratories, USA) was applied before imaging. Echocardiography measurements were acquired using a VisualSonics Vevo 3100 high frequency ultrasound imaging system (FUJIFILM VisualSonics, Canada) with a MX250 transducer (central transmit frequency: 21 MHz).

To enable LV cardiac functional measurements, echo kilohertz visualisation B-mode imaging of the Left ventricle was acquired in the parasternal long axis view (PLAX) for Fractional Area Change (FAC) measurements. M-mode images were acquired in a parasternal short axis (PSAX) view at the Mid-Papillary Level, with papillary muscles visible at the 2 and 4 o’clock positions for Fractional Shortening (FS) and Ejection Fraction (EF) measurements. Images were analysed using the Visualsonics VevoLab V5.9.0 image analysis software (FUJIFILM VisualSonics, Canada).

**Proteomics**

Approximately 100 to 250 mg of pooled tissue samples were extracted with lysis buffer (5% sodium dodecyl sulfate (SDS) in 50 mM triethylammonium bicarbonate buffer (TEAB)) in a ratio of 1:10 (w/v) and homogenised with ceramics beads (CK Mix Precellys ceramics beads; Bertin Technologies, France). After beads beating, each vial was centrifuged at 10,000 RCF for 10 min. Resulting supernatant was transferred into a new protein low binding vial, sonicated for 10 min (Bioruptor Picosonicator; Diagenode, Belgium) and centrifuged 10,000 RCF for 10 min. 500 µL of each clean supernatant were taken and protein concentration was determined using BCA assay (Thermo Scientific, USA). 30 µg of protein in 25 µL of lysis buffer were then reduced with 10 mM dithiothreitol (final concentration) for 1 h, 400 rpm at 37 °C and alkylated with 18.75 mM iodoacetamide for 35 min at room temperature in the dark. Reduced and alkylated supernatant were digested and washed with S-trap micro columns (ProtiFi, USA) with a high recovery protocol as described by the suppliers, adding three extra chloroform:methanol (200 µL, 1:1, v/v) washes to remove the lipid content and using Trypsin/Lys-C enzyme at an enzyme-substrate ratio of 1:20 for digestion. Peptides were eluted with 60 µL of 50 mM TEAB, followed by 60 µL of 0.1 % formic acid and 60 µL of 50 % acetonitrile in 0.1 % formic acid. Eluted peptides were dried in a SPD2010 SpeedVac (Thermo Scientific, USA) and cleaned up using C_18_ attractSPE spintips (Affinisep, Le Houlme, France) following manufacturer’s protocol. Purified peptides were separated on an Aurora-25 cm column (IonOpticks Australia) using an UltiMate RSLCnano LC System (ThermoFisher Scientific) coupled to a timsTOF HT mass spectrometer (Bruker) via a CaptiveSpray ionisation source. Tryptic digest was delivered to a trap column (Acclaim PepMap100, 5 μm, 100 Å, 100 μm i.d. × 2cm) at a flow rate of 20 μL/min in 100% solvent A (0.1% formic acid in LCMS grade water). After 4 min of loading and washing, peptides were transferred to the Aurora-25 analytical column and separated at a flow rate of 200 nL/min using a 60-min gradient from 7% to 35% solvent B (solvent B, 0.1% formic acid in acetonitrile). The column temperature was set at 50°C. For DDA-PASEF acquisition, the full scans were recorded from 100 to 1700 m/z spanning from 1.45 to 0.65 Vs/cm2 in the mobility (1/K0) dimension. Up to 10 PASEF MS/MS frames were performed on ion-mobility separated precursors, excluding singly charged ions which are fully segregated in the mobility dimension, with a threshold and target intensity of 1750 and 14,500 counts, respectively.

Raw mass spectral data was processed using PEAKS Studio X-Pro Software (Bioinformatics Solutions Inc., Canada). Search was performed against UniProt rat reference proteome (Rattus norvegicus) sequence database containing 22,866 entries with MS1 precursor tolerance of 20 ppm and MS2 tolerance of 0.06 Da. Fully-tryptic digestion allowing one missed cleavage, fixed modification of cystine (+57.02) and oxidation of methionine and deamination of asparagine and glutamine were specified as variable modification for database search. Peptide spectrum matches, peptides, and proteins were validated at a 1.0 % FDR using the decoy hit distribution. Quantitative LFQ analysis was performed and with optional ID transfer enabled. Following investigation of several normalisation approaches, protein expression relative to GAPDH was found to be the most appropriate by investigating other validated internal housekeeping markers (HPRT1 [4], Park7 [5]). In addition, GAPDH has also been shown to be the most stable protein for rat cardiac tissue [4]. Missing data points were imputed and statistical changes were calculated as previously described [6]. Differentially expressed up and down-regulated proteins were identified using a fold change cut-off of 4 and p<0.05. These protein hits then underwent functional enrichment analysis to summarise the gene ontology (GO) terms in WebGestalt 2019 [7] using over-representation analysis and the following parameters: Minimum number of IDs in the category= 5, Maximum number of IDs in the category= 2000, FDR Method= BH, Significance Level= FDR < 0.05, redundancy reduction using affinity propagation and weighted set cover. Z score heat maps were generated in R using the heatmap.2 function within gplots. For TSPO cluster analysis across all heart groups, the row dendrograms (proteins) were cut at a height of 5 in order to generate the top ~500 proteins clustered with TSPO. These proteins were then also assessed by functional enrichment analysis using the same parameters described above.

**Supplemental Results:**

| **Group** | **Timepoint** | **Region** | ***K_1_*** | | | ***BP_TC_*** | | |
| --- | --- | --- | --- | --- | --- | --- | --- | --- |
|  |  |  | **Mean** | **SD** | **Datapoints** | **Mean** | **SD** | **Datapoints** |
| **Naïve** | **d2** | Global Left Ventricle | 3.38 | 1.21 | 9 | 0.64 | 0.64 | 9 |
|  |  | Apical Anterior | 4.06 | 1.20 | 9 | 0.63 | 0.52 | 9 |
|  |  | Mid-Inferior | 4.20 | 0.86 | 9 | 0.44 | 0.35 | 9 |
| **Sham** | **d2** | Global Left Ventricle | 3.06 | 0.89 | 6 | 1.25 | 0.70 | 4 |
|  |  | Apical Anterior | 3.05 | 1.43 | 6 | 2.84 | 3.22 | 5 |
|  |  | Mid-Inferior | 4.00 | 1.61 | 6 | 1.24 | 0.89 | 3 |
|  | **d7** | Global Left Ventricle | 3.17 | 1.09 | 4 | 0.89 | 0.46 | 4 |
|  |  | Apical Anterior | 3.39 | 1.50 | 4 | 1.63 | 1.19 | 4 |
|  |  | Mid-Inferior | 3.62 | 1.48 | 4 | 0.53 | 0.16 | 4 |
|  | **d14** | Global Left Ventricle | 3.14 | 1.36 | 6 | 1.29 | 1.61 | 6 |
|  |  | Apical Anterior | 3.19 | 1.48 | 6 | 0.74 | 0.37 | 4 |
|  |  | Mid-Inferior | 3.77 | 1.94 | 6 | 1.04 | 1.11 | 6 |
|  | **d28** | Global Left Ventricle | 3.92 | 2.08 | 3 | 1.10 | 0.47 | 3 |
|  |  | Apical Anterior | 3.84 | 2.27 | 3 | 0.97 | 0.75 | 2 |
|  |  | Mid-Inferior | 4.86 | 3.10 | 3 | 0.51 | 0.28 | 2 |
| **MI** | **d2** | Global Left Ventricle | 2.59 | 0.88 | 4 | 1.12 | 0.74 | 4 |
|  |  | Apical Anterior | 1.01 | 0.29 | 4 | 4.47 | 3.17 | 4 |
|  |  | Mid-Inferior | 4.00 | 1.99 | 4 | 1.08 | 0.74 | 4 |
|  | **d7** | Global Left Ventricle | 2.71 | 0.99 | 5 | 0.91 | 0.45 | 5 |
|  |  | Apical Anterior | 1.30 | 0.79 | 5 | 2.41 | 1.85 | 5 |
|  |  | Mid-Inferior | 3.38 | 1.10 | 5 | 0.66 | 0.42 | 5 |
|  | **d14** | Global Left Ventricle | 2.34 | 0.84 | 5 | 0.76 | 0.60 | 4 |
|  |  | Apical Anterior | 1.22 | 0.55 | 5 | 1.20 | 0.22 | 3 |
|  |  | Mid-Inferior | 3.01 | 1.47 | 5 | 0.51 | 0.54 | 4 |
|  | **d28** | Global Left Ventricle | 3.75 | 1.46 | 3 | 0.41 | 0.21 | 2 |
|  |  | Apical Anterior | 1.29 | 0.73 | 3 | 1.01 | 1.35 | 3 |
|  |  | Mid-Inferior | 2.42 | 0.21 | 3 | 0.66 | 0.14 | 2 |

**Supplementary Table 1. Summary of kinetic modelling constants *K_1_* and *BP_TC_***

**Supplementary Figure 1. [^18^F]LW223 SUV_MBF_ analysis pipeline. a)** A dynamic PET heart crop file is created for cardiac [^18^F]LW223 analysis. The example shown is from the myocardial infarction cohort. **b)** Using the 17 segment AHA polarmap approach, myocardial blood flow (MBF) is then calculated from the first 4 min of dynamic data using de Grado modelling, and an average SUV is calculated between 70‒100 min. **c)** The resulting *SUV_70‒100_* 3D parameter map is then divided by the MBF parameter map in order to calculate **d)** *SUB_MBF_*, shown here as a polarmap.

**Supplementary Figure 2. Comparison of myocardial blood flow analysis and 2TCM generated output parameters. a)** MBF plotted against *K_1_* estimates **b)** SUV_MBF_ plotted against *BP_TC_* using all cohorts, timepoints and cardiac VOIs. **** indicates p ≤ 0.0001 for Pearson correlation.

**Supplementary Figure 3. Longitudinal Cardiac PET with [^18^F]LW223 in the sham cohort across all segments. a)** The [^18^F]LW223 signal in the naïve cohort (equivalent to day 0) was compared to the sham cohort for segment 1, **b)** segment 2, **c)** segment 7, **d)** segment 8, **e)** segment 13, **f)** segment 14, **g)** segment 17, **h)** segment 3, **i)** segment 4, **j)** segment 9, **k)** segment 10, **l)** segment 15, **m)** segment 5, **n)** segment 6, **o)** segment 11, **p)** segment 12, **q)** segment 16. All results are shown and the mean or mean± SEM. For all panels, naïve n=10, sham day 2 n=6, sham day 7 n=6, sham day 14 n=6, sham day 28 n=5. * indicates p ≤ 0.05, ** indicates p ≤ 0.01, *** indicates p ≤ 0.001 and **** indicates p ≤ 0.0001 for one-way ANOVA with post-hoc Dunnett’s vs. the naïve cohort. Data within segments 4 and 10 did not conform to Gaussian distribution and therefore instead a Kruskal-Wallis test with a Dunn’s post-hoc comparison was performed. **r)** Summary polar plot diagram, highlighting in blue, segments which are statistically different from the equivalent naïve region at any timepoint, with **s)** showing differences at day 2 and **t)** showing differences at day 28 post surgery.

**Supplementary Figure 4. Longitudinal Cardiac PET with [^18^F]LW223 in the MI cohort across all segments. a)** The [^18^F]LW223 signal in the naïve cohort was compared to the MI cohort for segment 1, **b)** segment 2, **c)** segment 7, **d)** segment 8, **e)** segment 13, **f)** segment 14, **g)** segment 17, **h)** segment 3, **i)** segment 4, **j)** segment 9, **k)** segment 10, **l)** segment 15, **m)** segment 5, **n)** segment 6, **o)** segment 11, **p)** segment 12, **q)** segment 16. All results are shown and the mean or mean± SEM. For all panels, naïve n=10, MI day 2 n=4, MI day 7 n=5, MI day 14 n=5, MI day 28 n=3. * indicates p ≤ 0.05, ** indicates p ≤ 0.01, *** indicates p ≤ 0.001 and **** indicates p ≤ 0.0001 for one-way ANOVA with post-hoc Dunnett’s vs. the naïve cohort. Data within segments 1, 7, 4, 10, 5, 6 and 12 did not conform to Gaussian distribution and therefore instead a Kruskal-Wallis test with a Dunn’s post-hoc comparison was performed. **r)** Summary polar plot diagram, highlighting in blue, segments which are statistically different from the equivalent naïve region at any timepoint, with **s)** showing differences at day 2 and **t)** showing differences at day 28 post surgery.

**Supplementary Figure 5. A higher [^18^F]LW223 signal within a segment at day 2 is predictive of a high segmental signal at day 28.** Correlation analysis for day 2 vs. day 28, containing all 17 segments from the MI cohort, n=3, ** indicates p ≤ 0.01 for Pearson correlation. LAD= left anterior descending artery territory, LCX= left circumflex artery territory, RCA= right coronary artery territory.

**Supplementary Figure 6. Longitudinal Cardiac [^18^F]LW223 PET showing statistically significant segments when comparing sham and MI cohorts. a)** Sham vs. MI comparison for segment 1, **b)** segment 2, **c)** segment 7, **d)** segment 8, **e)** segment 13, **f)** segment 14, **g)** segment 17, **h)** segment 3, **i)** segment 4, **j)** segment 9, **k)** segment 10, **l)** segment 15, **m)** segment 5, **n)** segment 6, **o)** segment 11, **p)** segment 12 and **q)** segment 16. All panels are shown as the mean± SEM, and sham day 2 n=6, sham day 7 n=6, sham day 14 n=6, sham day 28 n=5, MI day 2 n=4, MI day 7 n=5, MI day 14 n=5, MI day 28 n=3. * indicates p ≤ 0.05, ** indicates p ≤ 0.01, *** indicates p ≤ 0.001 and **** indicates p ≤ 0.0001 for sham vs. MI using a two-way ANOVA.

**Supplementary Figure 7. Average [^18^F]LW223 SUV_MBF_ cardiac polar plots comparrison between the reperfusion model, and the permannet ligation model.** The data for permannent ligation was calculated using our previously published data [1]. Both datasets are from 7 days after MI. The reperfusion plot is an average of 5 rat hearts, and the permanent ligation plot is an average of 8 rat hearts.

**Supplementary Figure 8. Low power magnification overview of histology in the sham and MI cohorts. a)** Representative examples of H&E staining across the sham and MI cohorts with the infarcts evident within the anterolateral walls. **b)** CD68 (red), TSPO (green) and DAPI nuclear counterstain (blue) shown by immunofluorescence in sections which are serial to the examples shown in panel A. Scale bars= 1mm. The white boxes show the approximate sampling location for the high-power magnification examples shown in Figure 2.

**Supplementary Figure 9. TSPO and CD68 immunofluorescent staining shown as individual channels.** Scale bars= 50 µm.

**Supplementary Figure 10. TSPO and CD68 immunofluorescent staining controls. a)** A representative H&E stain taken from an animal within the sham cohort. **b)** Using a serial section, the lack of autofluorescence was shown by omission of the TSPO and CD68 primary antibodies. **c)** TSPO and CD68 isotype control antibody staining. **d)** Opal antibody detection system crossover test were the TSPO primary antibody was omitted showing no cross reactivity between the second amplification step and the first amplification following destruction of the peroxidase by the mid antigen retrieval step. Scale bar= 1mm. In panels B-D, blue= nuclei, green=TSPO and red= CD68.

**Supplementary Figure 11. TSPO expression within cardiomyocytes increases with age in naïve rat hearts. a)** TSPO (green), CD68 (red) and DAPI nuclear counterstain (blue) staining shown in hearts sampled from four different naïve rats age-matched to the Sham/MI day 2 timepoint. **b)** The same staining shown in hearts sampled from four different naïve rats age-matched to the Sham/MI day 28 timepoint.

**Supplementary Figure 12. Summary of the percentage TSPO^+^CD68^+^ cells relative to the total amount of TSPO^+^ cells within the infarct/anterolateral wall across the different cohorts and timepoints. a)** **TSPO^+^CD68^+^** summary for naïve at day 2, **b)** naïve at day 28, **c)** sham at day 2, **d)** sham at day 7, **e)** sham at day 14, **f)** sham at day 28, **g)** MI at day 2, **h)** MI at day 7, **i)** MI at day 14, **j)** MI at day 28. The absolute number of cells, rounded to 0 decimal places, is shown within the brackets in each segment. Green= TSPO^+^, Red=TSPO^+^CD68^+^.

**Supplementary Figure 13. Summary of Go Terms. a)** Full list of Go Terms from the significantly upregulated proteins between d2 MI and d2 sham infarct/anterolateral regions. **b)** Full list of Go Terms from the significantly upregulated proteins between d28 MI and d28 sham infarct/anterolateral regions.

**Supplementary Figure 14. Functional systolic outcome measures assessed by ultrasound at day 28. a)** Comparison of sham MI cohorts for ejection fraction, **b)** fractional shortening, **c)** fractional area change. For all panels, the results are shown as the mean± SEM, with n=4 for the sham and n=5 for MI cohorts. * indicates p ≤ 0.05 and ** indicates p ≤ 0.01 for an unpaired t-test.

**Supplementary Figure 15. Ejection Fraction assessed by ultrasound correlates with Troponin I.** *** indicates p≤ 0.001 for Pearson correlation.

**Supplementary Figure 16. Individual comparison of additional cardiac ultrasound outcome measures and [^18^F]LW223 signal within the infarct/anterolateral wall. a)** Fractional shortening compared with the PET signal at day 2, **b)** day 7, **c)** day 14 and **d)** day 28. **e)** Fractional area change (FAC) compared with the PET sign at day 2, **f)** day 7, **g)** day 14 and **h)** day 28. n=4 for the sham and n=3-5 for MI cohorts, * indicates p ≤ 0.05, ** indicates p ≤ 0.01 and ns indicates p > 0.05 for Pearson correlation.

**Supplementary References:**

1. MacAskill MG, Stadulyte A, Williams L, Morgan TEF, Sloan NL, Alcaide-Corral CJ, et al. Quantification of Macrophage-Driven Inflammation During Myocardial Infarction with 18 F-LW223, a Novel TSPO Radiotracer with Binding Independent of the rs6971 Human Polymorphism. J Nucl Med [Internet]. 2021;62:536–44. Available from: http://jnm.snmjournals.org/lookup/doi/10.2967/jnumed.120.243600

2. MacAskill MG, Wimberley C, Morgan TEF, Alcaide-Corral CJ, Newby DE, Lucatelli C, et al. Modelling [18F]LW223 PET data using simplified imaging protocols for quantification of TSPO expression in the rat heart and brain. Eur J Nucl Med Mol Imaging [Internet]. 2021;49:137–45. Available from: https://link.springer.com/10.1007/s00259-021-05482-1

3. DeGrado TR, Hanson MW, Turkington TG, Delong DM, Brezinski DA, Vallée J-P, et al. Estimation of myocardial blood flow for longitudinal studies with 13N-labeled ammonia and positron emission tomography. J Nucl Cardiol [Internet]. 1996;3:494–507. Available from: https://linkinghub.elsevier.com/retrieve/pii/S1071358196900598

4. Kim HJ, Na JI, Min BW, Na JY, Lee KH, Lee JH, et al. Evaluation of protein expression in housekeeping genes across multiple tissues in rats. Korean J Pathol. 2014;48:193–200.

5. Wiśniewski JR, Mann M. A proteomics approach to the protein normalization problem: Selection of unvarying proteins for MS-based proteomics and western blotting. J Proteome Res. 2016;15:2321–6.

6. Aguilan JT, Kulej K, Sidoli S. Guide for protein fold change and: P-value calculation for non-experts in proteomics. Mol Omi. 2020;16:573–82.

7. Wang J, Vasaikar S, Shi Z, Greer M, Zhang B. WebGestalt 2017: A more comprehensive, powerful, flexible and interactive gene set enrichment analysis toolkit. Nucleic Acids Res. 2017;45:W130–7.
